# Surgical Antimicrobial Prophylaxis in Neonates and Children Undergoing Neurosurgery: A RAND/UCLA Appropriateness Method Consensus Study

**DOI:** 10.3390/antibiotics11070856

**Published:** 2022-06-26

**Authors:** Susanna Esposito, Mino Zucchelli, Sonia Bianchini, Laura Nicoletti, Sara Monaco, Erika Rigotti, Laura Venditto, Cinzia Auriti, Caterina Caminiti, Elio Castagnola, Giorgio Conti, Maia De Luca, Daniele Donà, Luisa Galli, Silvia Garazzino, Stefania La Grutta, Laura Lancella, Mario Lima, Giuseppe Maglietta, Gloria Pelizzo, Nicola Petrosillo, Giorgio Piacentini, Simone Pizzi, Alessandro Simonini, Simonetta Tesoro, Elisabetta Venturini, Fabio Mosca, Annamaria Staiano, Nicola Principi, on behalf of the Peri-Operative Prophylaxis in Neonatal and Paediatric Age (POP-NeoPed) Study Group

**Affiliations:** 1 Pediatric Clinic, University Hospital, Department of Medicine and Surgery, University of Parma, 43126 Parma, Italy; bianchini.sonia@outlook.it (S.B.); laura.nicoletti@studenti.unipr.it (L.N.); s.monaco1410@gmail.com (S.M.); 2 Pediatric Neurosurgery, IRCCS Istituto delle Scienze Neurologiche di Bologna, 40138 Bologna, Italy; mino.zucchelli@isnb.it; 3 Pediatric Clinic, Azienda Ospedaliera Universitaria Integrata, 37134 Verona, Italy; erika.rigotti@aovr.veneto.it (E.R.); venditto.laura@gmail.com (L.V.); giorgio.piacentini@univr.it (G.P.); 4 Neonatology and Neonatal Intensive Care Unit, IRCCS Bambino Gesù Children’s Hospital, 00165 Rome, Italy; cinzia.auriti@opbg.net; 5 Research and Innovation Unit, University Hospital of Parma, 43126 Parma, Italy; ccaminiti@ao.pr.it (C.C.); gmaglietta@ao.pr.it (G.M.); 6 Infectious Diseases Unit, IRCCS Giannina Gaslini, 16147 Genoa, Italy; eliocastagnola@gaslini.org; 7 Pediatric ICU and Trauma Center, Fondazione Policlinico Universitario A. Gemelli IRCCS, 00165 Rome, Italy; giorgio.conti@unicatt.it; 8 Paediatric Infectious Disease Unit, Academic Department of Pediatrics, IRCCS Bambino Gesù Children’s Hospital, 00165 Rome, Italy; maia.deluca@opbg.net (M.D.L.); laura.lancella@opbg.net (L.L.); 9 Division of Paediatric Infectious Diseases, Department for Woman and Child Health, University of Padua, 35100 Padua, Italy; daniele.dona@unipd.it; 10 Infectious Diseases Unit, Meyer Children’s Hospital, 50139 Florence, Italy; luisa.galli@unifi.it (L.G.) elisabetta.venturini@meyer.it (E.V.); 11 Paediatric Infectious Diseases Unit, Regina Margherita Children’s Hospital, University of Turin, 10122 Turin, Italy; silvia.garazzino@unito.it; 12 Institute of Translational Pharmacology IFT, National Research Council, 90146 Palermo, Italy; stefania.lagrutta@cnr.it; 13 Pediatric Surgery, IRCCS Azienda Ospedaliera-Universitaria di Bologna, 40138 Bologna, Italy; mario.lima@unibo.it; 14 Pediatric Surgery Department, “Vittore Buzzi” Children’s Hospital, 20154 Milano, Italy; gloria.pelizzo@unimi.it; 15 UniCampus University, 00125 Rome, Italy; nicola.petrosillo@inmi.it; 16 Pediatric Anesthesia and Intensive Care Unit, Salesi Children’s Hospital, 60123 Ancona, Italy; pizzis@me.com (S.P.); dr.simonini@gmail.com (A.S.); 17 Division of Anesthesia, Analgesia and Intensive Care, Department of Surgical and Biomedical Sciences, University of Perugia, 06129 Perugia, Italy; simonettatesoro@gmail.com; 18 NICU, Fondazione IRCCS Ca’ Granda Ospedale Maggiore Policlinico, Department of Mother, Child and Infant, 20122 Milan, Italy; fabio.mosca@unimi.it; 19 Department of Translational Medical Science, Section of Pediatrics, University of Naples “Federico II”, 80138 Naples, Italy; staiano@unina.it; 20 Università degli Studi di Milano, 20122 Milan, Italy; nicola.principi@unimi.it

**Keywords:** antibiotics, craniotomy, head fracture, pediatric infectious diseases, neurosurgery, surgical antibiotic prophylaxis

## Abstract

Pediatric neurosurgery is a highly specialized branch of surgery in which surgical site infections (SSIs) are potentially serious complications that can also adversely affect a good surgical outcome, compromising functional recovery and, in some cases, even putting the patient’s life at risk. The main aim of this consensus document is to provide clinicians with a series of recommendations on antimicrobial prophylaxis for neonates and children undergoing neurosurgery. The following scenarios were considered: (1) craniotomy or cranial/cranio-facial approach to craniosynostosis; (2) neurosurgery with a trans-nasal-trans-sphenoidal approach; (3) non-penetrating head injuries; (4) penetrating head fracture; (5) spinal surgery (extradural and intradural); (6) shunt surgery or neuroendoscopy; (7) neuroendovascular procedures. Patients undergoing neurosurgery often undergo peri-operative antibiotic prophylaxis, with different schedules, not always supported by scientific evidence. This consensus provides clear and shared indications, based on the most updated literature. This work has been made possible by the multidisciplinary contribution of experts belonging to the most important Italian scientific societies, and represents, in our opinion, the most complete and up-to-date collection of recommendations on the behavior to be held in the peri-operative setting in this type of intervention, in order to guide physicians in the management of the patient, standardize approaches and avoid abuse and misuse of antibiotics.

## 1. Introduction

Pediatric neurosurgery is a highly specialized branch of surgery that deals with studying, diagnosing, treating and monitoring pathologies involving the central (CNS) and peripheral (PNS) nervous system and which arise in childhood or affect adolescents [1]. Signs and symptoms attributable to neurological problems that may require surgery or neurological investigations with surgical methods include the following: delays in motor or cognitive development; abnormal increase in the volume of the skull or, conversely, below-average growth; abnormal shape of the head; changes in behavior or movements; lack of motor coordination or slow reflexes; seizures, tremors, spasms and muscle stiffness; changes in mood or level of consciousness; lethargy; muscle weakness; difficulty in language skills [2]. Severe headaches, loss of sensation or hearing loss, dizziness or visual changes (double vision, strabismus) may occur more frequently in older children or adolescents. The most common reasons for neurosurgery in newborns, infants and adolescents are hydrocephalus, skull and cranial fossa malformations, cerebral and spinal neoplasms, hemangiomas and other intracranial vascular malformations, Chiari malformation complex, spina bifida and other congenital or acquired malformations of the cranial-spinal cord, epilepsy, head and brain trauma (also including the sport-related concussion), spinal cord and spinal canal trauma [3].

Surgical site infections (SSIs) in neurosurgery are potentially serious complications that can also adversely affect a good surgical outcome, compromising functional recovery and, in some cases, even putting the patient’s life at risk. In addition to being dangerous, SSIs also entail high costs due to the prolongation of hospitalization, the prolonged use of antibiotics and, frequently, the need for further surgery [4]. Overall prevalence of SSIs in pediatric neurosurgery ranges from 0.5% to 14% in the antibiotic era, whereas the rate of infectious complications was as high as 58.8% in the pre-antibiotic era [1]. No detailed data are available for several countries, including Italy. Risk factors for SSIs after neurosurgical procedures include the following: need of ventricular drains, cerebrospinal fluid leak, procedure duration of more than two to four hours, placement of heterologous material, concurrent or previous shunt infection, and emergency procedures [4]. Surgical antibiotic prophylaxis (SAP) in neurosurgery, when indicated, can have a significant impact on patient’s morbidity, mortality and healthcare associated costs [5]. On the other hand, if prescribed inappropriately, SAP could increase the risk of infections, due to antimicrobial-resistant pathogens [5].

Unfortunately, data available in the literature are extremely poor on SAP in neurosurgery, especially those regarding the pediatric population. SAP is usually needed in most of the neurosurgical procedures and the scientifically validated guidelines for the prevention of SSIs are based on data and experiences in adult patients. However, the pediatric neurosurgery practice differs from that of adults treating children in various stages of physical and psychological development including diseases that do not exist in adults [6]. The main aim of this consensus document is to provide clinicians with a series of recommendations on antimicrobial prophylaxis for neonates and children undergoing neurosurgery.

## 2. Methods

### 2.1. RAND/UCLA Appropriateness Method

This consensus document was realized using the Research and Development Corporation (RAND, Santa Monica, CA, USA) and the University of California, Los Angeles (UCLA, Los Angeles, CA, USA) appropriateness method. The RAND/UCLA method consists of the appropriateness evaluation of diagnostic and therapeutic procedures with suboptimal scientific evidence by a panel of experts [7]. According to the RAND method, a procedure is defined as “appropriate” if the expected benefits outweigh the expected negative consequences, with a wide margin that justifies it, regardless of the costs. In contrast, a procedure whose expected risks outweigh the expected benefits is considered “inappropriate”. According to the RAND definition, experts who make an appropriateness/inappropriateness judgment must consider the clinical benefits and not be influenced by economic considerations. Therefore, appropriateness is used to evaluate the risk/benefit ratio of a list of diagnostic, management and therapeutic procedures [8]. For a heterogeneous topic such as surgical antimicrobial prophylaxis on which randomized controlled trials in pediatrics are lacking, the application of methods aiming to increase the homogeneity of behaviors by neonatologists, infectious diseases specialists, pediatric surgeons, and anesthetists appeared useful and appropriate. For this reason, the RAND/UCLA approach was chosen instead of GRADE methodology. Through the RAND method, the participants discussed different clinical scenarios and elaborated statements on the basis of the literature and their clinical experience. The group of experts did not consider it appropriate to combine the GRADE method with the RAND/UCLA approach because the absence of randomized studies represents a bias in defining the strength of the recommendations and in representing a consensus reached for real-life.

### 2.2. Recruitment of Panelists

A multidisciplinary group of experts belonging to the main Italian scientific societies dealing with anti-infective therapy of children was selected. The following Scientific Societies were involved: Italian Society of Pediatrics (SIP), Italian Society of Neonatology (SIN), Italian Society of Pediatric Infectious Diseases (SITIP), Italian Society of Infectious and Tropical Diseases (SIMIT), Italian Society of Pediatric Surgery (SICP), Italian Society of Microbiology (SIM), Italian Society of Pharmacology (SIF), Pediatric Pharmacology Study Group, Italian Society of Anaesthesia and Neonatal and Paediatric Resuscitation (SARNEPI), and Italian Society of Childhood Respiratory Diseases (SIMRI). The panel of experts comprised 52 medical doctors with at least 5 years’ experience: pediatricians (*n* = 20), neonatologists (*n* = 6), infectious diseases specialists (*n* = 5), pediatric surgeons including neurosurgeons (*n* = 5), anesthetists (*n* = 8), pharmacologist (*n* = 5) and microbiologists (*n* = 3).

### 2.3. Generation of Scenarios

Initially, literature search was performed with a selection of documents, including randomized studies, systematic reviews of the literature, meta-analyses and guidelines on perioperative prophylaxis for the prevention of SSI during plastic surgery. The literature search was carried out on the PubMed database, with a choice of articles in English published from 2000 until 2020. The key search terms were: “antimicrobial prophylaxis” OR “antibiotic prophylaxis” AND “neurosurgery” OR “craniotomy” OR “trans-nasal-transphenoidal surgery” OR “head fracture” OR “spinal surgery OR “shunt surgery” OR “neuroendovascular procedures” AND “neonate” OR “newborn” OR “paediatric” OR “pediatric” OR “children” OR “adolescent”. Subsequently, using the Patient/Problem/Population-Intervention-Comparison/Control/Comparator-Outcome (PICO) model, a questionnaire was created on perioperative prophylaxis during neurosurgical procedures in neonatal and pediatric patients, which were divided into 7 clinical scenarios. Before administration, it was tested twice with a one-week interval to a convenience sample of 4 pediatricians, 2 neonatologists, one infectious diseases specialist, one neurosurgeon, one anesthetist, one pharmacologists and one microbiologist. Then, 26 out of 52 experts were selected by the Scientific Societies for answering and the questionnaire was administered to 11 pediatricians, 3 neonatologists, 2 infectious diseases specialists, 3 pediatric surgeons including neurosurgeons, 4 anesthetists, 2 pharmacologists, and one microbiologist.

### 2.4. Two-Round Consensus Process

Based on the scenarios, the questionnaire was submitted to experts on the online platform REDCap. REDCap (Research Electronic Data Capture) is a secure, web-based software platform designed to support data capture for research studies, providing the following: (1) an intuitive interface for validated data capture; (2) audit trails for tracking data manipulation and export procedures; (3) automated export procedures for seamless data downloads to common statistical packages; (4) procedures for data integration and interoperability with external sources. Each question included the clinical scenario and possible answers were whether or not SAP was recommended for the scenario and, in case of its recommendation, a list with all the antibiotics available on the EU market so that the expert could select the antibiotics that he/she considered as first choice. The selected bibliographic material was made available to all panel members, who were instructed on how to fill out the questionnaire. The experts answered anonymously to the questionnaire, and their judgement was expressed on a 1–9 scale, where “1” was considered definitely inappropriate, “5” was considered uncertain, and “9” was considered definitely appropriate. Intermediate values corresponded to different modulations of the judgement of inappropriateness (“2” and “3”), uncertainty (from “4” to “6”) and appropriateness (“7” and “8”). In evaluating each indication, each expert referred both to their own experience and clinical judgement and to the available scientific evidence. A free space was provided for any annotation or comment.

The first round of the questionnaire was blinded to the other panel members. Multiple participation was not permitted by the platform, which also guaranteed the confidentiality and anonymity of the answers. The results of the survey were discussed in a collegial meeting with all the 26 experts who answered the questionnaire to reach agreements and reduce eventual disagreements. Clarifications, adaptations, and refinements of the indications and appropriateness ratings were made. A total of 7 recommendations were developed. Participants were asked to approve the recommendations in a second round during the following four weeks.

## 3. Results

### 3.1. SCENARIO #1. Craniotomy and Cranial/Cranio-Facial Approach to Craniosynostosis

It has been reported that craniotomy, albeit uncommonly, can be associated with the development of SSIs, such as epidural empyema, meningitis, subdural empyema or cerebral abscess [9,10,11,12,13]. Similarly, in craniosynostosis, defined as premature fusion of one or more cranial sutures with an estimated prevalence of 3 to 7.2 per 10,000 live births [14], a tailored treatment approach is of paramount importance, and risk of SSIs can vary from 0.8% to 7% according to the type of surgery and patient characteristics [15]. *Staphylococci* are the most common etiologic agents in epidural infections. In subdural infections, a role is also played by Gram-negative rods, including *Enterobacter* spp., *Pseudomonas* spp., and *Serratia* spp. [16]. To prevent these potentially life-threatening complications, SAP has been suggested since the first years of the antibiotic era [17]. However, its use was initially debated, as in some studies, a significant benefit of SAP was not demonstrated [18,19,20]. Further studies reinforced the idea that prophylactic antibiotics were necessary to protect patients undergoing craniotomy from SSIs. Effect on meningitis development has been the most frequently studied problem. A meta-analysis of studies published before October 2014 including 7 randomized controlled trials has shown that SAP was associated with a 66% reduction in meningitis development (odds ratio [OR] 0.35, 95% confidence interval [CI] 0.18–0.63) [21], confirming what had been reported in a previous meta-analysis (OR 0.43, 95% CI 0.20–0.92) [22]. However, no definitive conclusions could be drawn regarding both the time and duration of antibiotic administration, the choice of the most effective antibiotic, and the relevance of surgery duration in conditioning prophylaxis effect. Studies were too heterogeneous to allow reliable comparisons. Frequency of antibiotic administration varied from a single pre-operative dose to 6 doses. Prescribed antibiotics varied from drugs with spectrum limited to Gram-positive rods (vancomycin, oxacillin, cloxacillin) to drugs with extended spectrum including Gram-negative bacteria (cefotiam, piperacillin). Surgery duration varied from 107 to 312 min. Some more information regarding the best time for the antibiotic administration and the most suitable drug for SSI prevention in patients undergoing craniotomy can be derived by Cao et al. [23]. These authors performed an indirect comparison of efficacy between different antibiotic prophylaxis against SSIs after craniotomy, analyzing the clinical data of 3214 patients enrolled in 11 studies. They found that a single preoperative dose was adequate to substantially reduce the incidence of SSIs, with the exception of fusidic acid, for which no benefit was evidenced. All the other i.v. prescribed drugs (cefazolin, cefazedone, cefotiam, clindamycin, vancomycin, oxacillin, cloxacillin and piperacillin) had a positive, although slightly different, effect. Compared to no prophylactic antibiotic or placebo, clindamycin was the most effective drug (OR 0.09, 95% CI 0.01–071, *p* = 0.02) and cephalosporins considered together with the less effective (0R 0.35, 95% CI 0.21–0.59, *p* = 0.01). Despite some of the studies included in this analysis having significant methodological limitations suggesting the need for further, well conducted trials, the authors concluded that antibiotic prophylaxis with a single antibiotic preoperative dose was mandatory and that reduced-spectrum antibacterial drugs could be used. On the other hand, suggestions like these have been developed by various American and European scientific societies [24,25,26]. In most of the cases, cefazolin was suggested as the drug of choice with clindamycin and vancomycin as alternatives to be used in case of beta-lactam allergy or when the patient carries MRSA, respectively. Considering the increasing number of MRSA both in the hospital and in the community, we recently suggested that in the patient undergoing neurosurgery, in an emergency or elective regimen, although routine screening for *Staphylococcus aureus* nasal colonization is not recommended, this may be strongly suggested in cases at high-risk of MRSA infection (i.e., those with MRSA preoperative colonization, those with a history of MRSA infection, neonates, and infants less than three months of age who have been hospitalized since birth or have a complex heart disorder) [3]. In the neonatal or pediatric patients colonized by MRSA, performing decolonization in the preoperative phase is recommended, using mupirocin nasal ointment at one application in each nostril three times a day and also a shower a day with soapy chlorhexidine (or povidone iodine for patients for whom chlorhexidine is contraindicated) for 5 days before surgery [3,27]. Recent literature suggests that also nasal methicillin-susceptible *S. aureus* (MSSA) decolonization with mupirocin should be performed because it reduces the rate of post-operative SSIs especially in cardiac surgery, orthopedic surgery as well as in neurosurgery [28]. Although decolonization of *Staphylococci* could not be reached using nasal mupirocin, it can reduce bacterial burden. Despite this, if decolonization treatment is performed, as we explained in our previous study [3], we recommend only cefazolin as SAP. In case of colonization by MRSA or MSSA, or if the patient cannot wait 5 days for decolonization pre-surgery, vancomycin in addition to cefazolin is recommended for SAP [29]. Due to the pharmacokinetics of vancomycin, it is recommended to end its infusion 60 min before skin cut to allow vancomycin to reach optimal drug distribution in blood and tissue [30]. Moreover, vancomycin infusion should not be too fast and should last 30–60 min to prevent red men syndrome and other side effects [31]. This means that vancomycin should be administered 90–120 min before surgery, whereas cefazolin should be given as the last drug during anesthesia induction just before skin cut.

Several aspects of SAP in patients undergoing craniotomy remain unsolved. One of the most important is whether patients at increased risk of developing cerebrospinal fluid (CSF) leakage after craniotomy must receive long-term antibiotic prophylaxis. CSF leakage has been identified as a significant risk factor for meningitis [32] and, as meningitis develops several hours after craniotomy, it seems likely that the single dose preoperative prophylaxis may be insufficient to inhibit bacterial replication in CSF. A second problem is whether antibiotic currently recommended for prophylaxis can reach into the CSF adequate concentrations to prevent meningitis, particularly in patients having non-inflamed meninges. Vancomycin, for example, has variable but generally poor ability to penetrate CSF [33]. Studies specifically devoted to preventing SSIs in neonates and children undergoing craniotomy are lacking and our panel of experts agreed to follow recommendations suggested for adults.

**Recommendation 1.** In neonates and children undergoing craniotomy or cranial/cranio-facial approach to craniosynostosis, administration of cefazolin at a dose of 30 mg/kg (maximum dose 2 g) IV administered 30 min before surgery is recommended. A further dose after 4 h should be given when the procedure lasts for a long time. In patients colonized by MRSA or MSSA who did not perform decolonization pre-surgery, or if the patient cannot wait 5 days for decolonization pre-surgery, cefazolin at a dose of 30 mg/kg (maximum dose 2 g) IV combined with vancomycin at a dose of 15 mg/Kg (maximum dose 2 g) IV is recommended. Cefazolin should be administered 30 min before surgery, whereas vancomycin is recommended 90–120 min before.

### 3.2. SCENARIO #2. Neurosurgery with a Trans-Nasal-Trans-Sphenoidal Approach

Endoscopic endonasal trans-sphenoidal surgery is a minimally invasive, well-tolerated procedure presently considered the preferred approach for several brain diseases, mainly pituitary tumors [34] and, more recently, for other more complex conditions located in the midline brain and in the lateral skull base [35]. As it passes through the nasal cavity to reach the sterile brain spaces, it is considered a clean-contaminated procedure. Bacteria that colonize the nose can be transferred to the sphenoidal sinus and the subarachnoid space leading to the development of bacterial infections. Sinusitis and meningitis are relatively common in patients undergoing this procedure, as they have been reported in 0.5–14% and in 3.6–9.6% of the cases [36]. Moreover, this procedure can be associated with CSF leakage and subsequent meningitis, even several months later than the surgical procedure. In a study enrolling 98 adult cases 11 CSF leaks (11%) and 10 CNS infections (10%) were evidenced [37]. Immediately postoperative infections are mainly due to Gram-positive bacteria, especially *Streptococcus* spp. and *Staphylococcus* spp., whereas in meningitis following CSF leakage Gram-negative rods are frequently encountered.

To avoid the risk of postsurgical SSI development, perioperative antibiotic prophylaxis is commonly prescribed in patients undergoing endoscopic trans-sphenoidal surgery. A survey carried out by Little and White among the membership of the International Society of Pituitary Surgeons has shown that 81% of them used SAP [38]. However, true efficacy of SAP is far to be demonstrated and, as evidenced in the survey, about 90% of surgeons are aware that there is a lack of high-quality evidence supporting antibiotic prescription. Results of studies evaluating antibiotic prophylaxis in neurosurgery with a trans-nasal-trans-sphenoidal approach are conflicting, with many in favor of antibiotics and others that do not report effectiveness compared to placebo or no treatment. A systematic review of the studies published until December 2018 revealed that available literature was generally limited and of very low quality. However, due to the variety of the antibiotic regimens used and the predominant observational study design these studies could not be used to perform a meta-analysis. Moreover, no definitive conclusions were available regarding which drugs could offer the best protective effect and how and when they should be administered. This accounts for the lack of official recommendation by scientific societies. However, while waiting for randomized controlled trials able to solve all the presently unsolved problems, it can be highlighted that recent studies indicate that a short-term antibiotic regimen (cefazolin up to 24 h) or an ultrashort-term antibiotic regimen (ampicillin-sulbactam, a single intraoperative dose) can be effective in reducing the risk of SSIs, mainly meningitis, in patients undergoing endonasal trans-sphenoidal surgery [39,40,41]. These regimens could represent a reasonable compromise between the desire to reduce the risk of infectious complications and the need to avoid useless antibiotic prescriptions. No specific data are available for children for whom recommendations similar to those for adults can be followed. In addition, as for craniotomy, screening for *Staphylococcus aureus* nasal colonization could be suggested and preoperative decolonization in case of MRSA recommended.

**Recommendation 2.** In neonates and children undergoing neurosurgery with a trans-nasal-trans-sphenoidal approach, administration of cefazolin at a dose of 30 mg/kg (maximum dose 2 g) IV administered 30 min before surgery is recommended. A further dose after 4 h should be given when the procedure lasts for a long time. In patients colonized by MRSA who did not perform MRSA decolonization pre-surgery, cefazolin at a dose of 30 mg/kg (maximum dose 2 g) IV combined with vancomycin at a dose of 15 mg/Kg (maximum dose 2 g) IV is recommended. Cefazolin should be administered 30 min before surgery, whereas vancomycin is recommended 90–120 min before.

### 3.3. SCENARIO #3. Neurosurgery in Non-Penetrating Head Fracture

Head trauma is a common cause of morbidity and mortality in children, sometimes resulting in a bone fracture. Although most pediatric cranial fractures can be treated conservatively, some require a surgical approach, especially those involving the frontal bones [42] or resulting in a deeply depressed skull [43]. Data regarding the efficacy of SAP in patients undergoing surgery for the correction of a skull fracture after non-penetrating head injury are almost absent in the literature. To date, there are no clear recommendations, and the few data available are extrapolated from the adult population. Most authors recommend routine use of SAP in order to reduce the risk of infective complications. Others instead suggest prophylactic antibiotic therapy only in particular cases of high-risk fractures, such as fractures of the skull base and frontal bones, as they are at a higher risk of CSF leakage and therefore to infectious complications [44]. Our expert panel reached the consensus on routine SAP recommendation with cefazolin in non-penetrating head fracture.

**Recommendation 3.** In neonates and children undergoing neurosurgery for non-penetration head injuries, administration of cefazolin at a dose of 30 mg/kg (maximum dose 2 g) IV administered 30 min before surgery is recommended. A further dose after 4 h should be given when the procedure lasts for a long time.

### 3.4. SCENARIO #4. Neurosurgery in Penetrating Head Fracture

Infectious complications, mainly local wound infections, meningitis, ventriculitis, or cerebral abscess, occur in 1–11% of patients with penetrating brain injuries and can significantly increase the risk of prolonged hospital stay, persistent neurological impairment and death [45]. The risk is greater in case of air sinus wounds, CSF leakage, transventricular injuries or when foreign objects, skin, hair, and bone fragments enter the brain tissue [46,47]. *Staphylococcus aureus* is the most frequently associated organism, although Gram-negative aerobe rods and anaerobes can be detected [45]. Antibiotic prophylaxis has been advocated since long time, but in 2001, the British Society for Antimicrobial Chemotherapy after evaluation of available studies concluded that data in published reports were insufficiently complete to provide guidance on antimicrobial prophylactic use [48]. However, as in the pre-antibiotic era the rate of infectious complications was as high as 58.8% and SAP had been found able to reduce this value significantly, it was recommended that all the patients undergoing neurosurgery for penetrating head injuries received prophylaxis with antibiotics. Large spectrum drugs given as soon as possible and continued for 5 days after surgery were suggested. This is because it was thought that an antibiotic highly effective against *Staphylococcus aureus* but with relatively poor efficacy against Gram-negative rods could be inadequate. In particular, i.v. amoxicillin-clavulanic acid or i.v. cefuroxime in association with i.v. metronidazole were indicated. With the appropriate adjustments according to the weight, the same prophylaxis was suggested for children. In the following years, despite some new studies, no definitive conclusion with regard to antibiotic selection, dose, and duration was drawn. Recommendations previously reported remain valid, although in some studies, a significant reduction in the duration of antibiotic administration was not associated with an increase in infectious complication incidence. In the study by Marut et al., some patients received only the preoperative dose, and some others were given drugs for no more than 24 h [49]. Due to the lack of pediatric data, suggestions made for adults can be followed also for children.

**Recommendation 4.** In neonates and children undergoing neurosurgery for penetrating head fracture, administration of amoxicillin-clavulanic acid at a dose of 30 mg/kg (maximum dose 1 g) IV or cefuroxime at a dose of 50 mg/kg (maximum dose 1.5 g) IV in association with metronidazole at a dose of 15 mg/kg (7.5 mg/kg in neonates weighing less than 1200 g; maximum dose 500 mg) IV is recommended. Administration should begin within 30 min before surgery and it is recommended for 5 days.

### 3.5. SCENARIO #5. Spinal Surgery (Extradural and Intradural)

Despite spinal surgery being a clean surgery, risk of postoperative SSIs development is not marginal. In adults, studies have shown that it can vary from less than 1% to 15% depending on several surgical- and patient-related risk factors, such as the type and duration of the procedure, nutritional status, immunosuppression, and comorbidities [50]. To reduce this risk, several prophylactic measures have been suggested and studied in clinical practice. Among them, intra-wound vancomycin powder administration, closed-suction drainage, povidone-solution irrigation, 2-octyl-cyanocrylate skin closure and perioperative antibiotic administration. Unfortunately, many studies have very low quality, and this explains why results of these studies are frequently conflicting and firm conclusions on the real role of each of these prophylactic measures cannot be drawn. However, taking into account the available data, it seems possible to conclude that, among topical measures, intra-wound vancomycin powder administration and povidone-iodine irrigation seem to play the major role in reducing risk of SSIs development after both non-instrumented and complex instrumented spinal procedures. Salimi et al. showed that intra-wound vancomycin had no effect on SSIs; in addition, it can increase the rate of Gram-negative infections [51]. A systematic review reported that available data indicated there was moderate and limited evidence for the efficacy of the povidone-iodine irrigation and the intra-wound vancomycin administration, respectively [52]. More recent studies have confirmed at least in part these findings [53]. A study involving 853 adult patients reported that subjects treated with the povidone-iodine irrigation compared to control patients experienced a significant reduction in the incidence of superficial SSIs (risk ratio [RR] 0.18; 95% CI 0.04–0.80), although incidence of deep SSIs was similar (RR 1.00; 95% CI 0.57–1.73). In the vancomycin group, incidence of both superficial (RR 0.31; 95% CI 0.12–0.81) and deep (RR 0.52; 95% CI 0.29–0.92) SSIs was significantly lower than in the control group [53]. Finally, a meta-analysis published in 2021 evidenced that both the vancomycin (odds ratio [OR] 0.53; 95% CI 0.39–0.71) and the povidone-iodine (OR 0.10; 95% CI 0.04–0.23) were significantly more efficacious than the placebo in SSI reduction [54]. Regarding SAP, Yao et al. concluded, on the basis of a few randomized controlled trials and some retrospective studies, that there was fair evidence that a single preoperative antibiotic dose could be effective in reducing SSI risk in patients undergoing spinal surgery, regardless of the type of procedure and patient characteristics [52].

Further antibiotic doses for one or more days after the end of the surgical procedure were not associated with a significant benefit and, on the contrary, could increase the risk of drug-related adverse events and emergence of resistant bacterial strains. Several studies have shown that the incidence of SSIs is quite similar in patients receiving only a preoperative dose, two days of postoperative antibiotics or longer prophylaxis [53,54,55]. On the other hand, the few studies showing the superiority of a prolonged antibiotic prophylaxis, at least in instrumented spine procedure, had in most of the cases several methodological limitations [55,56]. In the only one randomized trial, patients with extended antibiotic administration had a slightly lower incidence of SSIs compared to patients with only the preoperative antibiotic dose (1.7% vs. 4.3%) [55]. However, the study group consisted of only 269 cases, and the differences in rates between groups did not reach statistical significance. Finally, in a recent, prospective non-randomized cohort study enrolling 5208 patients, it was found that SSIs incidence was 5.3% in patients receiving the single preoperative dose and 2.2% in those given antibiotics for 72 h (*p* < 0.01) [56]. Unfortunately, this study had several limitations, including the fact that patients with long-term prophylaxis were compared to patients with preoperative prophylaxis enrolled several years before. This explains why all the scientific societies that have prepared guidelines for antibiotic prophylaxis in spine surgery recommend only to use preoperative drug administration [57,58]. Such recommendation is also shared by the North American Spine Society (NASS), which suggests prolonged postoperative regimens in complex situations (trauma, cord injury, neuromuscular disease, diabetes or other comorbidities) [59]. Regarding drug choice, all the guidelines highlight that there are no sufficient data to establish which is the most effective drug, dose, and route of administration to obtain the best protection from SSIs and that the patient’s risk factors, allergies, length and complexity of the procedure, and issues of antibiotic resistance should drive the selection of the potentially effective drug. Redosing during procedure depends mainly on pharmacokinetic characteristics of the prescribed drugs. Finally, NASS recommend to consider, particularly in complex instrumented cases, the use of drugs effective against Gram-negative rods and/or the application of intrawound vancomycin or gentamicin. This is because, in complicated instrumented cases, about 10% of patients can have a polymicrobial SSI [60]. Despite guidelines not indicating a drug of choice, most of the studies have been carried out with first and second generation cephalosporins with clindamycin as an alternative for patients with beta-lactam allergy and vancomycin for those living in areas with high incidence of MRSA. Among cephalosporins, cefazolin i.v. given about 30 min before skin incision, with a second administration in case of surgery lasting more than 4 h, appeared the most common prescription.

Studies evaluating the efficacy of antimicrobial prophylaxis in children undergoing spinal procedures are lacking. It has been established that the risk for SSIs in children is quite similar to that found in adults, varying from 3.5% to 5.2%, with the highest values in children requiring instrumental surgery [61,62]. Cefazolin, vancomycin, or clindamycin were the most commonly used drugs in studies including children [63,64].

**Recommendation 5.** In neonates and children undergoing spine surgery, regardless of the type of surgical procedure, administration of cefazolin at a dose of 30 mg/kg (maximum dose 2 g) IV administered 30 min before surgery is recommended. A further dose after 4 h should be given when the procedure lasts for a long time. In patients colonized by MRSA or MSSA who did not perform decolonization pre-surgery, or if the patient cannot wait 5 days for decolonization pre-surgery, cefazolin at a dose of 30 mg/kg (maximum dose 2 g) IV combined with vancomycin at a dose of 15 mg/Kg (maximum dose 2 g) IV is recommended. Cefazolin should be administered 30 min before surgery, whereas vancomycin is recommended 90–120 min before.

### 3.6. SCENARIO #6. Shunt Surgery and Neuroendoscopy

Shunt surgery, including ventriculoperitoneal shunt, ventriculoatrial shunt and cyst peritoneal shunt surgery, is a most common measure generally used to treat hydrocephalus. As with shunt surgery, a foreign body is implanted, and risk of SSIs is high. Studies have shown that incidence of SSIs after shunt surgery can vary from 1% to 39%, with a higher prevalence in children than in adults and, among children, in premature and younger infants, in those with post-infectious hydrocephalus, and in those with a previous shunt infection [65,66,67]. Moreover, SSIs can be associated with prolonged hospitalization, need for shunt revision or removal and, albeit rare, death [68].

*Staphylococci* are the most common infecting pathogens. To reduce SSI incidence after shunt surgery, antibiotic prophylaxis has been largely used both in adults and children. Antibiotics were given before, during or after surgery or in various combinations. Different routes of administration were used: oral, i.v., directly into the brain or the shunt or using antibiotic-impregnated catheters. Results of studies are conflicting and, although they generally indicate that prophylaxis reduces the risk of SSIs, no definitive conclusions regarding the efficacy of the different routes of administration, the time and duration of prophylaxis as well as the choice of the antibiotic can be drawn [69,70,71,72,73,74]. A Cochrane review, including only 11 randomized controlled trials published before January 2018 and a total of 1109 participants, concluded that antibiotic prophylaxis was significantly effective, as patients undergoing shunt surgery who had received antibiotics had a 45% reduction in rate of SSIs compared to patients receiving standard care or placebo (RR 0.55; 95% CI 0.36–0.84) [75]. However, as most of the studies were at high risk of bias, evidence of this review was considered of very low certainty. Moreover, only i.v. administration showed a statistically significant effect, whereas for the other methods of antibiotic administration, the number of available studies was too low to allow a reliable evaluation.

Several doubts regarding antibiotic prophylaxis of shunt surgery remain unsolved. Among them are whether antibiotic ability to penetrate the blood–brain barrier has an impact on the efficacy of i.v. antibiotic administration and whether the poor passage can condition the administration of a combined i.v. or intrathecal therapy or the use of an antibiotic-impregnated catheter. However, despite these limitations suggesting the need for further studies, many authors prefer the administration of i.v. antibiotics only before surgery, with redosing of the drug after some hours, according to its pharmacokinetic characteristics, when the duration of the shunt surgery exceeds normal duration. No post-operative doses are considered mandatory. Contrarily to most of the other surgical procedures, shunt procedures have been studied in the pediatric population. Pediatric neurosurgeons suggest cefazolin as the drug of choice, with vancomycin or clindamycin as alternative in case the procedure is performed in areas with high MRSA circulation [76].

Neuroendoscopy is now often used as the gold standard for the treatment of many conditions once treatable only with shunts [77,78,79]. Although there are no specific pediatric studies, the panel recommends the same SAP indications for neuroendoscopy as for shunts.

**Recommendation 6.** In neonates and children undergoing shunt surgery or neuroendoscopy, administration of cefazolin at a dose of 30 mg/kg (maximum dose 2 g) IV administered 30 min before surgery is recommended. A further dose after 4 h should be given when the procedure lasts for a long time.

### 3.7. SCENARIO #7. Neuroendovascular Procedures

The most common complications of neuroendovascular procedures are ischemic stroke and intracranial and hemorrhagic; on the contrary, intracranial infections are exceptional, as clearly evidenced by Kelkar et al. [80]. These authors reviewed 2918 cerebral angiograms and neurointerventional procedures conducted without prophylactic antibiotics and found that there were only 3 infections (0.1%) attributable to the procedure [80]. On the other hand, Burkhardt et al., who conducted a retrospective study comparing patients who received SAP with cefazolin with others that did not receive any prophylaxis, did not show a difference in terms of post-operative infections between the two groups [81]. These findings indicate that the routine use of antibiotic prophylaxis in patients undergoing neuroendovascular procedures is useless and cannot be recommended. Despite the lack of specific studies in children, there are no reasons to think that they should receive prophylaxis when they undergo neuroendovascular procedures.

**Recommendation 7.** Antibiotic prophylaxis is not recommended in children undergoing neuroendovascular procedures.

## 4. Discussion

For the greatest part of the neurosurgical procedures, no official guidelines developed by qualified scientific societies are available for neonates and children. This depends on the lack of controlled clinical trials defining which is the real risk of infection for each surgical procedure, what is the true impact of antibiotic administration on infection incidence, which is the drug of choice and how and when it must be administered. Unfortunately, very few studies are available, and for several surgical procedures, no definitive conclusions can be drawn. This leads neurosurgeons to prescribe antibiotics very frequently because they fear the consequences of an infection rather than because of the real effectiveness of antibiotic prophylaxis and leads them to use a schedule of antibiotic administration frequently based on personal criteria instead of demonstrated efficacy. Moreover, even when the use of antibiotic prophylaxis seems to be scientifically supported and the choice of the drug is based on reliable data, several aspects of antibiotic administration remain unsolved. In particular, the duration of the antibiotic prophylaxis is not established for many neurosurgical procedures. This is a relevant point as to reduce the risk of adverse event development and the emergence of antibacterial resistance. Moreover, some peculiar aspects of infections in neurosurgery have only been marginally considered. This is the case in craniotomy and shunt surgery, in which meningitis is the main infectious complication and in which antibiotics suggested for prophylaxis cannot reach adequate concentration in the CSF. In the case of shunt surgery, it is not clarified whether impregnated catheters are really effective and can be the only anti-infective procedure to prevent meningitis. In addition, these data evidence how many studies are urgently needed because antibiotic prophylaxis in neurosurgery can be effective for the patient and for the health system. This is extremely important considering the peculiarities of the neonate and the child in the first years of life, because all the suggestions inevitably follow what is proposed for the adult.

In our scenarios, we discussed the role of nasal colonization due to MRSA or MSSA [28,29]. This is a topic still debated, and in the case of colonization or if the patient cannot wait 5 days for decolonization, pre-surgery vancomycin in addition to cefazolin is recommended for SAP. We also discussed the different pharmacokinetics of vancomycin and cefazolin, with the practical implications for SAP administration [31].

One topic that represents a cross for neurosurgeons, and one that we did not include among the scenarios, is represented by external ventricular drainages [82]. Since this is a situation in which there is direct intra-/extra-blood–brain barrier communication, and since the liquor is difficult to “contain”, the risk of SSIs is very high, and the management is complex. For some years, there have been antibiotic-impregnated catheters that should reduce the risk of contamination or, at least, reduce the need for peri-operative antibiotics [83]. In fact, the risk of contamination is extremely low in the absence of CSF fistula and, so far, no increase in antibiotic resistance has been observed with antibiotic-impregnated catheters. With these premises, preoperative cefazolin can be used in older patients in whom it is possible to use catheters impregnated with antibiotic and carry out long tunneling to reduce the risk of CSF fistula; in neonates (especially in those low-birth-weight), if it is not possible to use medicated catheters, and when the CSF fistula is almost certain, the combination with vancomycin is the most suitable strategy [84,85]. However, further studies are needed to clarify the optimal SAP and the effectiveness of catheters impregnated with antibiotic when external ventricular drainages are positioned.

Through the RAND method, in our study, the participants discussed the statements and the agreement was reached in the recommendations. A literature review is presented in detail in each scenario. It should be noted that the participants in the project came from different clinical contexts, i.e., they were pediatricians, neonatologists, infectious diseases specialists, pediatric neurosurgeons, pediatric surgeons, anesthetists, pharmacologists and microbiologists. For this reason, the results achieved demonstrate the usefulness of the RAND method for the selection of good practices and constitute the basis of an evidence-based approach. The findings obtained can establish the basis for educational interventions that aim to optimize the use of antibiotics in pediatric patients undergoing neurosurgical procedures. Limitations of the study included that this was an opinion-based survey and the agreement was reached on a collegial meeting. On the other hand, the lack of pediatric studies on the topic prevented the use of the GRADE methodology, and the complexity of the topic required an online face-to-face meeting with all the participants.

This consensus document aimed to respond to issues that are still little addressed, with the ambition to fill current shortcomings. The specific scenarios developed are intended to guide the healthcare professional in practice to ensure a better and standardized management of the neonatal and pediatric patient. Table 1 summarizes the seven recommendations for antibiotic prophylaxis in neonatal and pediatric neurosurgery.

## 5. Conclusions

Patients undergoing neurosurgery are considered special patients for the risk of developing SSIs that can lead to severe CNS complications. They often undergo peri-operative antibiotic prophylaxis, with different schedules, not always supported by scientific evidence. This consensus provides clear and shared indications, based on the most updated literature.

This work represents, in our opinion, the most complete and up-to-date collection of recommendations on the behavior to be held in the peri-operative setting in this type of intervention, in order to guide physicians in the management of the patient, standardize approaches and avoid abuse and misuse of antibiotics [86]. Undoubtedly, more randomized and controlled trials are needed in the pediatric population to better define the best therapeutic management and real usefulness of the antibiotic prophylaxis.

## Figures and Tables

**Table 1:** Recommendation of antibiotic prophylaxis in neonatal and pediatric neurosurgery.

Type of Neurosurgical Procedure	Recommendation
Craniotomy or cranial/cranio-facial approach to craniosynostosis	Cefazolin at a dose of 30 mg/kg (maximum dose 2 g) IV administered 30 min before surgery is recommended. A further dose after 4 h should be given when the procedure lasts for a long time. In patients colonized by MRSA or MSSA who did not perform decolonization or if the patient cannot wait 5 days for decolonization pre-surgery, cefazolin at a dose of 30 mg/kg (maximum dose 2 g) IV combined with vancomycin at a dose of 15 mg/Kg (maximum dose 2 g) IV is recommended. Cefazolin should be administered 30 min before surgery, whereas vancomycin is recommended 90–120 min before.
Neurosurgery with a trans-nasal-trans-sphenoidal approach	Cefazolin at a dose of 30 mg/kg (maximum dose 2 g) IV administered 30 min before surgery is recommended. A further dose after 4 h should be given when the procedure lasts for a long time. In patients colonized by MRSA who did not perform MRSA decolonization pre-surgery, cefazolin at a dose of 30 mg/kg (maximum dose 2 g) IV combined with vancomycin at a dose of 15 mg/Kg (maximum dose 2 g) IV is recommended. Cefazolin should be administered 30 min before surgery, whereas vancomycin is recommended 90–120 min before.
Neurosurgery in non-penetrating head injuries	Cefazolin at a dose of 30 mg/kg (maximum dose 2 g) IV administered 30 min before surgery is recommended. A further dose after 4 h should be given when the procedure lasts for a long time.
Neurosurgery in penetrating head fracture	Amoxicillin-clavulanic acid at a dose of 30 mg/kg (maximum dose 1 g) IV or cefuroxime at a dose of 50 mg/kg (maximum dose 1.5 g) IV in association with metronidazole at a dose of 15 mg/kg (7.5 mg/kg in neonates weighing less than 1200 g; maximum dose 500 mg) IV is recommended. Administration should begin within 30 min before surgery and is recommended for 5 days.
Spinal surgery (extradural and intradural)	Cefazolin at a dose of 30 mg/kg (maximum dose 2 g) IV administered 30 min before surgery is recommended. A further dose after 4 h should be given when the procedure lasts for a long time. In patients colonized by MRSA or MSSA who did not perform decolonization pre-surgery or if the patient cannot wait 5 days for decolonization pre-surgery, cefazolin at a dose of 30 mg/kg (maximum dose 2 g) IV combined with vancomycin at a dose of 15 mg/Kg (maximum dose 2 g) IV is recommended. Cefazolin should be administered 30 min before surgery, whereas vancomycin is recommended 90–120 min before.
Shunt surgery or neuroendoscopy	Cefazolin at a dose of 30 mg/kg (maximum dose 2 g) IV administered 30 min before surgery is recommended. A further dose after 4 h should be given when the procedure lasts for a long time.
Neuroendovascular procedures	Not recommended

## Data Availability

All the data are included in the manuscript.
